# Characterization of three novel genetic loci encoding bacteriocins associated with *Xanthomonas perforans*

**DOI:** 10.1371/journal.pone.0233301

**Published:** 2020-05-29

**Authors:** Mizuri Marutani-Hert, Aaron P. Hert, Simone M. Tudor-Nelson, James F. Preston, Gerald V. Minsavage, Robert E. Stall, Pamela D. Roberts, Sujan Timilsina, Jason C. Hurlbert, Jeffrey B. Jones

**Affiliations:** 1 Department of Plant Pathology, University of Florida, Gainesville, Florida, United States of America; 2 Microbiology and Cell Sciences, University of Florida, Gainesville, Florida, United States of America; 3 Southwest Florida Research and Education Center, University of Florida, Immokalee, Florida, United States of America; 4 College of Arts and Sciences, Winthrop University, Rock Hill, South Carolina, United States of America; Technical Educational Institute of Peloponnese, GREECE

## Abstract

Bacterial spot is a destructive disease of tomato in Florida that prior to the early 1990s was caused by *Xanthomonas euvesicatoria*. *X*. *perforans* was first identified in Florida in 1991 and by 2006 was the only xanthomonad associated with bacterial spot disease in tomato. The ability of an *X*. *perforans* strain to outcompete *X*. *euvesicatoria* both *in vitro* and *in vivo* was at least in part associated with the production of three bacteriocins designated Bcn-A, Bcn-B, and Bcn-C. The objective of this study was to characterize the genetic determinants of these bacteriocins. Bcn-A activity was confined to one locus consisting of five ORFs of which three (ORFA, ORF2 and ORF4) were required for bacteriocin activity. The fifth ORF is predicted to encode an immunity protein to Bcn-A based on *in vitro* and *in vivo* assays. The first ORF encodes Bcn-A, a 1,398 amino acid protein, which bioinformatic analysis predicts to be a member of the RHS family of toxins. Based on results of homology modeling, we hypothesize that the amino terminus of Bcn-A interacts with a protein in the outer membrane of *X*. *euvesicatoria*. The carboxy terminus of the protein may interact with an as yet unknown protein(s) and puncture the *X*. *euvesicatoria* membrane, thereby delivering the accessory proteins into the target and causing cell death. Bcn-A appears to be activated upon secretion based on cell fractionation assays. The other two loci were each shown to be single ORFs encoding Bcn-B and Bcn-C. Both gene products possess homology toward known proteases. Proteinase activity for both Bcn-B and Bcn-C was confirmed using a milk agar assay. Bcn-B is predicted to be an ArgC-like serine protease, which was confirmed by PMSF inhibition of proteolytic activity, whereas Bcn-C has greater than 50% amino acid sequence identity to two zinc metalloproteases.

## Introduction

Bacterial spot of tomato is incited by four *Xanthomonas* species: *X*. *euvesicatoria*, *X*. *vesicatoria*, *X*. *perforans*, and *X*. *gardneri* [1]. The first two species, *X*. *euvesicatoria* and *X*. *vesicatoria*, were previously reported to be extensively distributed worldwide [1]. *X*. *gardneri*, which was recently reclassified as a pathovar of *X*. *cynarae*, was first reported from former Yugoslavia and has been since reported from USA, Canada, Brazil, Ethiopia, and Reunion [2–6]. Meanwhile, *X*. *perforans* is an emerging pathogen with a worldwide distribution affecting major tomato production areas [7].

In Florida, prior to 1991, only *X*. *euvesicatoria* was present on tomatoes in Florida, but in 1991, *X*. *perforans* was first identified in those same fields [8]. In fields where both *X*. *euvesicatoria* and *X*. *perforans* were present, *X*. *perforans* became predominant over a single season [8]. In an extensive survey in Florida conducted in 2006, 377 strains were isolated from bacterial spot lesions in 20 tomato fields [9]; all strains were identified as *X*. *perforans*, indicating that this bacterium had displaced *X*. *euvesicatoria*. This phenomenon was determined to be due at least in part to production of bacteriocins by *X*. *perforans* strains that were toxic to *X*. *euvesicatoria* strains [10, 11].

Bacteriocins are generally proteinaceous toxins that are toxic towards closely related bacterial competitors [12]. Bacteriocins of gram-negative bacteria represent a diverse group of proteins in terms of size, microbial target, mode of action, and immunity mechanism. The most extensively studied bacteriocins are the colicins produced by *Escherichia coli* [13–19]. Relatively few reports are available on the production of bacteriocin compounds by phytopathogenic bacteria [20]. In the 1950s, Okabe *et al*. published the first article on phytopathogenic bacteria, in which strains of *Pseudomonas* (*Ralstonia*) *solanacearum* were inhibitory only to other *P*. *solanacearum* strains [21]. *Agrobacterium radiobacter* K84 was shown to produce a unique substituted analogue of adenosine, agrocin 84, toxic to other closely related strains [22]. Other examples can be found in plant pathogenic bacteria in various genera including *Erwinia*, *Clavibacter*, *Pseudomonas*, *and Ralstonia* [16, 23–26].

There have been few reports of xanthomonads producing bacteriocins [11, 27, 28]. In a study by Fett et al. [25], *Xanthomonas campestris* pv. *glycines* was shown to produce at least one bacteriocin, while there was evidence for production of multiple bacteriocins by some strains. In another study *X*. *campestris* pv. *glycines* strain 8ra was shown *to* produce glycinecin A against *X*. *vesicatoria* [29]. Glycinecin A is a heterodimer consisting of GlyA (39 kDa) and GlyB (14 kDa) subunits. The *glyC* gene product located upstream of *glyA* and *glyB* helps in the secretion of glycinecin A.

In *X*. *perforans*, Tudor-Nelson *et al*. identified bacteriocin activity by screening a genomic library in a sensitive strain [11]. Three groups of clones were identified that showed bacteriocin activity and represented three bacteriocin loci (i.e., Bcn-A, Bcn-B and Bcn-C), which were unique in activity based on *X*. *euvesicatoria-*sensitive strains [11]. The shifts in bacterial spot causing *Xanthomonas* population in Florida in large part has been attributed to the antagonistic activity of bacteriocins produced by *X*. *perforans* against *X*. *euvesicatoria* [30]. However, the bacteriocin associated loci were not functionally characterized. In this study, we report the cloning and characterization of the three bacteriocins, Bcn-A, Bcn-B, and Bcn-C. We confirmed that four genes are necessary for Bcn-A activity: ORFA (*bcnA*), ORF2, ORF3, ORF4; and a fifth gene, ORF5, is an immunity gene. We also show that *bcnB* is found on a second genetic locus and encodes a protein (Bcn-B) predicted to be a serine protease based on sequence analysis and confirmed by inhibition assays. The third genetic locus found in the *X*. *perforans* strain contained the *bcnC* gene which encodes a protein (Bcn-C) that is predicted to be a metalloprotease by amino acid sequence analysis.

## Materials and methods

### Bacterial strains and plasmids

The bacterial strains and plasmids used in this study are shown in Table 1 and the primers are listed in Table 2. *Xanthomonas* and *Escherichia coli* were grown as described previously [11].

**Table 1:** Bacterial strains and plasmids used in this study.

Strain or plasmid	Relevant characteristics^#^	Source or reference*
***Xanthomonas euvesicatoria***		
91–106	wild type Nal^R^	[11]
91–106Δ*xpsD*	91–106::Δ*xpsD* Nal^R^Cm^R^	[31]
91-106(pLAFR119)	Empty vector, Nal^R^Tc^R^	[11]
91-106(pXV519)	BcnA^+^, Nal^R^Tc^R^	[11]
91-106(pXV442)	BcnB^+^, Nal^R^Tc^R^	[11]
91-106(pXV120)	BcnC^+^, Nal^R^Tc^R^	[11]
91–106Δ*xpsD* (pLAFR119)	Δ*xpsD*, Nal^R^Cm^R^Tc^R^	[31]
91–106Δ*xpsD*(pXV8.0)	Δ*xpsD*, pXV8.0, BcnA^-^, Nal^R^Cm^R^Tc^R^	[31]
91–106 (pLAFR119-ORF5)	Δ*xpsD*, pLAFR119-ORF5, Nal^R^Cm^R^Tc^R^	[31]
91–106Δ*xpsD* (pLB5.8)	Δ*xpsD*, pLB5.8, BcnB^-^, Nal^R^Cm^R^Tc^R^	[31]
91–106Δ*xpsD* (pXV1.7)	Δ*xpsD*, pXV1.7, BcnC^-^, Nal^R^Cm^R^Tc^R^	[31]
ME-90	Wild-type 85-10Rif^R^Km^R^	[11]
ME-90 (pLAFR119)	Empty vector, Rif^R^Km^R^Nal^R^Tc^R^	[11]
ME-90 (pXV519)	BcnA^+^, Rif^R^Km^R^Tc^R^	[11]
ME-90 (pXV442)	BcnB^+^, Rif^R^Km^R^Tc^R^	[11]
ME-90 (pXV120)	BcnC^+^, Rif^R^Km^R^Tc^R^	[11]
***X*. *perforans***		
91-118^R^	Wild-type, Rif^R^	[10]
91–118ΔBcnA	BCNA^-^B^+^C^+^, Rif^R^Sp^R^	[30]
91–118ΔBcnB	BCNA^+^B^-^C^+^, Rif^R^Km^R^	[30]
91–118ΔBcnC	BcnA^+^B^+^C^-^, Rif^R^Cm^R^	
91–118ΔBcnBC	BCNA^+^B^-^C^-^, Rif^R^Km^R^Cm^R^	[30]
91–118 Transposon in orfA	Transposon inserted in orfA, ΔorfA, BcnA^-^	[11]
91–118 Transposon in orf4	Transposon inserted in orf4, Δorf4, BcnA^-^	[30]
91-118ΔORFA	91-118ΔORFA, Rif^R^	[31]
91-118ΔORF2	91-118ΔORF2, Rif^R^	[31]
91-118Δ ORF3	91-118ΔORF3, Rif^R^	[31]
91-118ΔORF4	91-118ΔORF4, Rif^R^	[31]
***Escherichia coli***		
DH5α	F^-^ recA	BRL
C2110	Nal^R^	BRL
λPIR	Host for pOK1; Nal^R^, *ori*R6K RK2 replicon	UB
RK2013	helper plasmid; Km^R^ Tra^*+*^	[30]
**Plasmids**		
pOK1	Suicide vector; Sp^R^, SacB	[32]
pOK1Δ*gumD*	Δ*gumD* fragment into pOK1, Sp^R^	[31]
pOK1Δorf2	Δ*orf2* fragment into pOK1, Sp^R^	[31]
pOK1Δorf3	Δ*orf3* fragment into pOK1, Sp^R^	[31]
pOK1Δorf4	Δ*orf4* fragment into pOK1, Sp^R^	[31]
pOK1Δ*xpsD*	Δ*xpsD* fragment into pOK1, Sp^R^Cm^R^	[31]
pBluescript® II KS^+^ (pBS)	Phagemid, pUC derivative; Amp^R^	Stratagene
pBS-ORF2	PCR product with primers orf2F and orf2R into pBS, Amp^R^	[31]
pBS-ORF3	PCR product with primers orf3F and orf3R into pBS, Amp^R^	[31]
pBS-ORF4	PCR product with primers orf4F and orf4R into pBS, Amp^R^	[31]
pBS-ORF5	PCR product with primers orf5F and orf5R into pBS, Amp^R^	[31]
pBS-ORF2,3	PCR product with primers orf2F and orf3R into pBS, Amp^R^	[31]
pBS-ORF2-5	PCR product with primers orf2F and orf5R into pBS, Amp^R^	[31]
pBSΔORF2	*Δorf2* fragment into pBS, Amp^R^	[31]
pBSΔORF4	*Δorf4* fragment into pBS, Amp^R^	[31]
pGEM^®^-T Easy (pGEM)	Cloning vector, Amp^R^	Promega
pGEM-*xpsD*	PCR product with primers xpsDF and xpsDR into pGEM, Amp^R^	[31]
pGEMΔ*orf3*	Δ*orf3* fragment into pGEM, Amp^R^	[31]
pGEMΔ*xpsD*	Δ*xpsD* fragment into pGEM, Amp^R^Cm^R^	[31]
pLAFR3	Tc^R^ *rlx*^*+*^ RK2 replicon	BJS
pLAFR119	pLAFR3 containing pUC119 polycloning site (*Hind*III to *Eco*RI)	[10]
pLAFR119-*orf2*	Subclone of *orf2* into pLAFR119	[31]
pLAFR119-*orf2*,*3*	Subclone of *orf2*,*3* into pLAFR119	[31]
pLAFR119-*orf2-5*	Subclone of *orf2-5* into pLAFR119	[31]
pLAFR119-*orf3*	Subclone of *orf3* into pLAFR119	[31]
pLAFR119-*orf4*	Subclone of *orf4* into pLAFR119	[31]
pLAFR119-*orf5*	Subclone of *orf5* into pLAFR119	[31]
pLAFR119-*orfA*	Subclone of *orfA* into pLAFR119	[31]
pXV519	pLAFR3 cosmid clone from 91–118 (BCNA^+^) (~29 kb)	[10]
pXV12.1	EcoRI subclone derived from pXV519 (12.1 kb) into pLAFR119 (BCNA+IMMA+)	[10]
pXV3.5	*BamH*I/*Kpn*I subclone (3.5 kb) derived from pXV12.1 into pLAFR119 (BCNA^+^IMMA^+^)	[10]
pXV4.5	*BamH*I/*Eco*RI subclone (4.5 kb) derived from pXV12.1 into pLAFR119 (BCNA^-^MMA^+^)	[10]
pXV4.8	*Sal*I/*Eco*RI subclone (4.8 kb) derived from pXV12.1 into pLAFR119 (BCNA^-^IMMA^-^)	[10]
pXV6.2	*BamH*I subclone (6.2 kb) derived from pXV12.1 into pLAFR119 (BCNA^-^IMMA^-^)	[10]
pXV8.0	KpnI/EcoRI subclone (8.0 kb) derived from pXV12.1 into pLAFR119 (BCNA+IMMA+)	[10]
pXV1.7	*Sal*I/*Eco*RI BCNC subclone of pXV5.1 in forward orientation in pLAFR119 (BCNC+)	[10]
pXV1.7CR	BCNC subclone of pXV5.1 in reverse orientation in pLAFR119 (BCNC-)	[31]
pXV442	Cosmid clone from 91–118 into pLAFR3 (BCNB^+^)	[10]
pXV8.9	*Kpn*I subclone (8.9-kb) derived from pXV442 into pLAFR3 (BCNB^+^)	[10]
pLB5.8	*Kpn*I/*Eco*RI subclone (5.8 kb) derived from pXV8.9 (BCNB^+^) pLAFR3 (BCNB+)	[30]
pLB3.0	KpnI/HimdIII subclone (3.0 kb) derived from pLB5.8 into pLAFR119 (BCNB+)	[31]
pLB3.0 stop	*Kpn*I/*Himd*III subclone (3.0 kb) derived from pLB5.8 with a TAA stop codon insertion into pLAFR119 (BCNB^-^)	[31]
pLB2.4	*Xba*I/*Himd*III subclone (2.3 kb) derived from pLB5.8 into pLAFR119 (BCNB^-^)	[31]
pLB2.3	*Kpn*I/*Himd*III subclone (2.4 kb) derived from pLB5.8 into pLAFR119 (BCNB^-^)	[31]
pXV120	cosmid clone from 91–118 into pLAFR3 (BCNC^+^)	[10]
pXV5.1	*Hin*dIII/*Eco*RI subclone (5.1-kb) derived from pXV120 into pLAFR119 (BCNC^+^)	[10]
pLC2.5	HindIII/EcoRV subclone (2.5kb) derived from pXV5.1 into pLAFR119 (BCNC-)	[31]

*BRL, Bethesda Research Laboratories, Gaithersburg, MD; Stratagene, Stratagene Inc., La Jolla, CA; Promega Corporation, Madison, WI; BJS, B. J. Staskawicz, University of California, Berkeley, CA; UB, U. Bonas, Martin-Luther-Universität, Halle, Germany.

^#^Rif, Rifamycin; Km, Kanamycin; Nal, Nalidixic acid; Amp, Ampicillin; Sp, Spectinomycin; Cm, Chloramphenicol.

**Table 2:** Sequence of primers in this study.

	Primer	Restriction site	Primer Sequence	Length	GC (%)	Tm (°C)
BcnA	A5	*Xho*I	CCTCGAGATGCGCCACCCGTCGG	16	75	60
	A3	*Xho*I	CCTCGAGCAGCAAAAGCTGATAGAGC	19	47.3	54
	ORF3F	*Hin*dIII	CCCGAAGCTTCCGGTTGACCTCTATGTAGATGGATGC	36	55.6	68.8
	ORF3R	*Hin*dIII	CCCGAAGCTTCCCAGTGCAAATGTAAGCCGCGAC	33	60.6	69.6
	ORF2F	*Hin*dIII	GGGGAAGCTTACACAGGACGGGACATGCACAG	31	61.3	68.9
	ORF2R	*Hin*dIII	GGGGAAGCTTACAACCTCCACATCTCGCACCG	31	61.3	68.9
	ORF4F	*Hin*dIII	CCCAAGCTTGCCGGATGCGACATTGTTGCGC	31	61.3	70.2
	ORF4R	*Hin*dIII	CCCAAGCTTGCTTGGTTCAAGCTCATCACC	30	53.3	66.5
	ORF5F	*Hin*dIII	GGGGAAGCTTCAGGGTGGCGGCAAGGGA	27	70.3	70.2
	ORF5R	*Hin*dIII	GGGGAAGCTTGGGCTTCTCTGGAAGCGGAC	29	65.5	69.5
	ORF5DF		GGGAGATCTCATCCATTATCCGCTCG	26	50	65.3
	ORF5DR		GGGCTTAAGCGCAGGACTGACTCCACAACC	30	56.7	67.8
BcnB	B5' new	*Eco*RI	CGGAATTCCAATCGCAAGAACGCGATG	21	50	63.4
	B32	*Kpn*I	CGGGTACCCTGGCCGAAGTAGGTGGAAAT	21	52.3	69.3
	BORF1F		ATGGGCTTGTCGGCCACATAATCGTCACAA	30	50	68.1
	BORF1R		TTGTGACGATTATGTGGCCGACAAGCCCAT	30	50	68.1
	BORF2F		AACGAACGAAGGTTACACTGGCTCCACCAT	30	50	68.1
	BORF2R		ATGGTGGAGCCAGTGTAACCTTCGTTCGTT	30	50	68.1
	BORF3F	Stop	ATGAATCGCAAGTAAGCGATGTATCTGGCG	30	46.7	66.8
	BORF3R		CGCCAGATACATCGCTTACTTGCGATTCAT	30	46.7	66.8
	BORF4F		ATGGCTGCAAATTGATAATGCGCTCACGGT	30	46.7	66.8
	BORF4R		ACCGTGAGCGCATTATCAATTTGCAGCCAT	30	46.7	66.8
	BORF5F		CAGCCCGCGCGATTAGATGACCATTGCCAT	30	56.7	70.9
	BORF5R		ATGGCAATGGTCATCTAATCGCGCGGGCTG	30	56.7	70.9
	BORF7F		ATGGACGATCGCTAACCGTCGATCCGCTTC	30	56.7	70.9
	BORF7R		GAAGCGGATCGACGGTTAGCGATCGTCCAT	30	56.7	70.9
	B5XhoI	*Xho*I	CCTCGAGATGAATCGCAAGAACGCG	18	52.3	58
BcnC	C5'	*Eco*RI	CGGAATTCCGTGAAGAACGTCTTCCTC	27	51.9	54
	C3'	*Kpn*I	GGGGTACCCTTGTCGTCATCGTTCTGCGCCGGAGTGTT	37	50	54
	C5XhoI	*Xho*I	CCTCGAGGTGAAGAACGTCTTCCTC	18	61.1	58
T2SS	xpsDF		ATGACGCCGCGCCTGTTTCC	20	65	58
	xpsDR		CCCTTCTCAAGTGGCTGCAT	20	60	58

### Bacteriocin antagonistic activity and immunity in *X*. *perforans*

The deferred antagonism assay [33] was performed to determine inhibitory and immunity activity to Bcn-A bacteriocin as described previously [11].

### Characterization of *bcnA* by mutagenesis and subcloning

**Fig 1:**
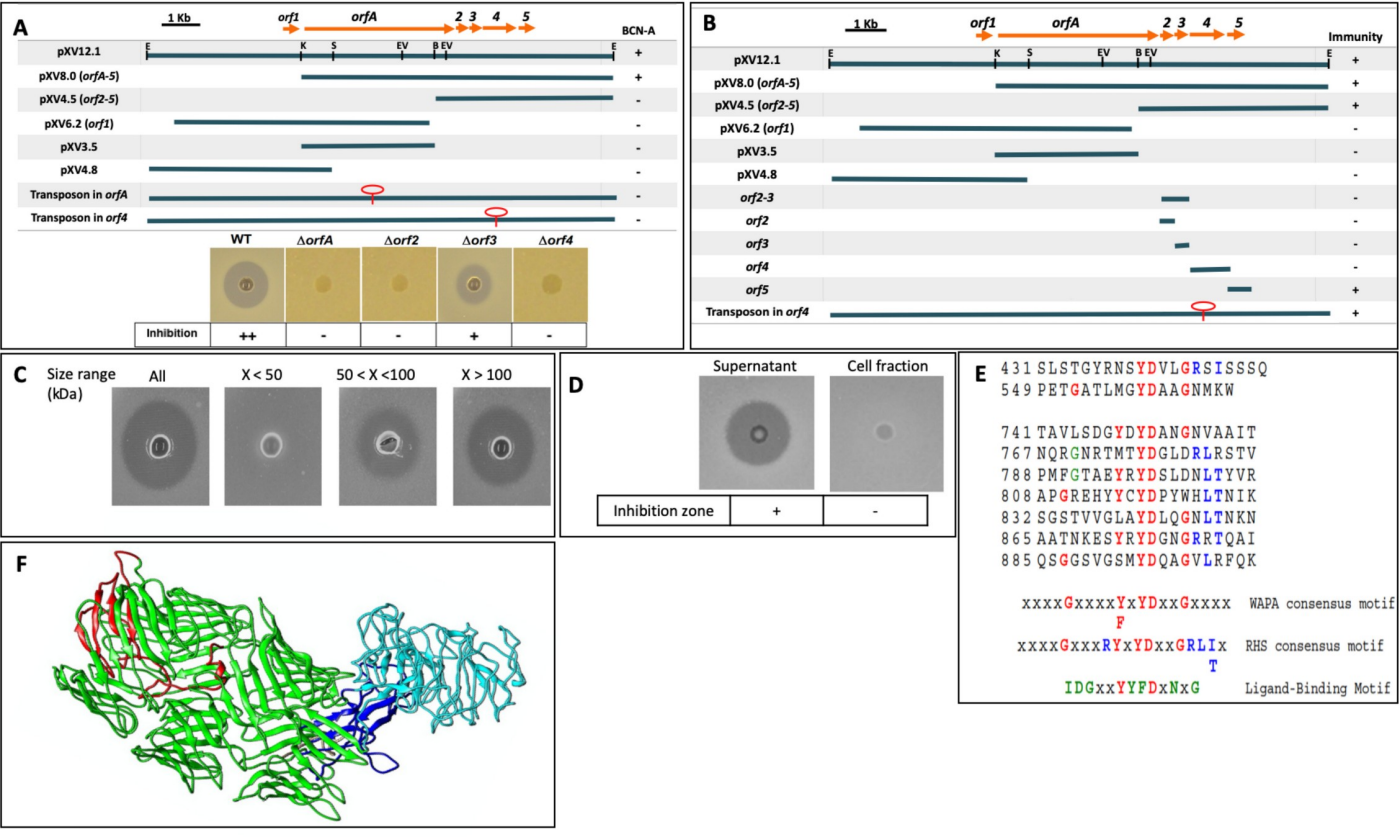
Identification of genes associated with Bcn-A and characterization of Bcn-A. (A) Schematic map of ORFs in pXV12.1 and Bcn-A activity when wild-type clone and deletion mutants of four ORFs were transformed into *X*. *euvesicatoria* 91–106 and tested against *X*. *euvesicatoria* 91–106. (B) Subcloning of Bcn-A gene cluster to identify immunity gene. Immunity function associated with ORF5. (C) Supernatant from *X*. *perforans* was separated based on size exclusion. Activity was associated mostly with size exclusion > 100 kDa. (D) Bcn-A activity is associated with supernatant but not with cell fraction. (E) Alignment of Bcn-A repeating motif region. Number represents position of Y in YD conserved sequence. X- Conserved in WAPA, RHSA, and ligand-binding consensus sequences; X- Only found in RHS consensus sequences; X- Only found in ligand-binding consensus sequences. (F) Lowest energy homology model of BcnA generated by iTasser. The amino terminal plug domain is colored blue, the beta-propeller domain is colored cyan, the YD-barrel is colored green and the carboxy-terminal region is colored red.

In previous work, Tudor [10] identified a cosmid clone, pXV519, which contained *bcnA* and was inhibitory to *X*. *euvesicatoria in vitro* and *in vivo* [11]. The insert from pXV519 was sub-cloned and designated pXV12.1 (Fig 1A), and contained the five genes necessary for Bcn-A expression. The clone was sequenced and consist of 10,513 bp (GenBank accession AF454545.2). The clone was further subcloned to an 8.0-kb fragment, designated pXV8.0, and contained six open reading frames (ORFs) identified in the sequenced region. A gene encoding a putative immunity protein against Bcn-A was mapped to a 4.5-kb *Bam*HI/*Eco*RI (pXV4.5) fragment downstream of ORFA [11].

Clones generated by PCR for each of the individual ORFs and some combinations were generated in pBluescript^®^ II KS (pBS) or pGEM^®^-T Easy (pGEM) (Promega, Madison, WI) for sequencing and transfer to pLAFR119 for screening for activity. To generate the bacteriocin mutants, genes for ORFA, ORF2,ORF3 and ORF4 were disrupted either by deletion or transposon mutagenesis to create 91–118ΔORFA, 91–118ΔORF2, 91–118ΔORF3, and 91–118ΔORF4. The ΔORFA mutant was constructed by deleting an *EcoR*V fragment from pXV8.0. All other mutants were created by using surrounding sequences up and down stream of the target ORF sequence. For construction of the 91-118ΔORF2 mutant, PCR was performed with primers A5 and A3, then the resulting ORFA PCR product was inserted upstream of ORF3 (primers ORF3F and ORF3R) subcloned in pBS to create pBSΔORF2. The fragment containing ORFA and ORF3 was subcloned into the suicide vector pOK1 with *Apa*I and *Xba*I to create pOK1ΔORF2 which was marker exchanged into 91–118 to create 91-118ΔORF2. To make 91-118ΔORF3, PCR was performed with primers ORF2F and ORF2R. The resulting PCR product was inserted upstream of ORF4 (primers ORF4F and ORF4R) in pGEM to create pGEMΔORF3. The fragment containing ΔORF3 was subcloned into suicide vector pOK1 with *ApaI* and *Sal*I creating pOK1ΔORF3. To make 91-118ΔORF4, PCR was performed with primers ORF2F and ORF3R, then the resulting PCR product was inserted upstream of ORF gene (primers ORF5F and ORF5R) in pBS-ORF5 to create pBSΔORF4. The pBSΔORF4 fragment was then subcloned into suicide vector pOK1 with restriction enzymes *Apa*I and *Xba*I and was mated into 91–118 to make the mutant. Mutants were confirmed by PCR. Each mutant strain was tested for bacteriocin activity against the sensitive *X*. *euvesicatoria* strain 91–106.

### Evaluation of Bcn-A immunity gene

An *in vitro* population assay was performed to evaluate immunity activity. A strain producing Bcn-A (91–118ΔBcnBC) was grown at 28°C overnight in 5 mL of NB with shaking. Cells were then pelleted, washed, resuspended in NB, standardized to 5x10^6^ colony-forming units (CFU)/mL, and incubated at 28°C with shaking. After incubating for 6 h, 5 × 10^5^ CFU/mL of the *X*. *euvesicatoria* 91–106 transconjugants with pLAFR119 or pLAFR119-ORF5 were added to each flask. Samples were assayed at 24 h intervals for 96 h. Each experiment was conducted three times and population data were transformed to logarithmic values that were averaged for the three replications and standard deviations were determined.

An *in vivo* population assay was performed to evaluate immunity activity. Bacterial strains were grown in nutrient broth for 18 h, harvested by centrifugation and resuspended in sterile tap water. The *X*. *perforans* 91–118ΔBcnBC and sensitive test *X*. *euvesicatoria* 91–106 (pLAFR119) or transconjugant 91–106 (pLAFR119-ORF5) strains were inoculated at 5x10^7^ CFU/mL and 5x10^6^ CFU/mL, respectively. The *X*. *perforans* strains were inoculated into leaflets by infiltration 18 h prior to inoculation with the sensitive test strain. Intercellular spaces of leaflets of six-week-old seedlings of the tomato cultivar Florida 47 were infiltrated (15 leaflets each per plant) using a hypodermic syringe as described previously [34]. Each treatment consisted of three replications. Following inoculation, plants were incubated at 24°C to 28°C. Bacterial populations were quantified in 1-cm^2^ leaf disks removed from the infiltrated area. The disks were macerated in 1 mL of sterile tap water and dilution plated onto NA amended with the appropriate antibiotics. Samples were incubated at 28°C and assayed at 24 h intervals from 48 to 96 h. Each experiment was conducted three times. Population data were transformed to logarithmic values and standard errors were determined.

### Identification of Bcn-B and Bcn-C genes

The insert of cosmid clone designated pXV442, containing *bcnB* [11, 35] was digested with *Kpn*I and *EcoR*1 and fragments were ligated into pLAFR119 to create a subclone designated pLB5.8 [31]. This insert was sequenced and submitted to GenBank (accession AB302849.1). pLB5.8 was conjugated into *X*. *euvesicatoria* ME90 and transconjugants were screened for bacteriocin activity against *X*. *euvesicatoria* strain 91–106. The possible gene upstream of *bcnB* was disrupted by adding an insertion stop codon (TAA) using a pair of primers (BORF3F and BORF3R) and a Quick-change XL Site Directed Mutagenesis kit (Stratagene, CA) (Fig 3A). Each transconjugant was tested for bacteriocin activity against the sensitive test strain *X*. *euvesicatoria* 91–106.

A cosmid clone, designated pXV120 that contains *bcnC* was originally identified by Tudor [35]. A 5.1-kb fragment containing *bcnC* was subcloned into pLAFR119 and designated pXV5.1 [10]. The insert was sequenced and submitted to Genbank (accession AB302850.1). Following digestion with *Sal*I/*EcoR*I, an insert from the clone was ligated into pLAFR119 and the subclone designated pXV1.7 was mobilized into *X*. *euvesicatoria* strain 91–106 (Fig 4A). Transconjugants were tested for bacteriocin activity against the wild-type *X*. *euvesicatoria* 91–106.

### Role of type II secretion system in delivery of bacteriocins

To determine the possible role of the type II secretion system (T2SS, formerly general secretion pathway, GSP) in the extracellular secretion of Bcn-A, Bcn-B, and Bcn-C, we created a deletion mutant to inactivate the T2SS protein, *gspD* (*xpsD*). In order to clone *xpsD* gene, primers xpsDF and xpsDR were designed using 91–118 genome sequence (Genbank assembly accession NZ_CP019725) in BlastQuest Genomelink by ICBR Bioinformatics’ System Group. PCR was performed using 91–118 genomic DNA as a template. A 2,229 bp *gpsD* gene was amplified and subcloned into pGEM. A chloramphenicol resistance cassette from pRKP10 was inserted in an internal *KpnI* site to disrupt *xpsD*. The disrupted *xpsD* gene was subcloned into suicide vector POK1 with *ApaI* and *SpeI*. The final plasmid construct was mated into 91–106 to create the type II secretion mutant *X*. *euvesicatoria* 91-106Δ*xpsD*, which was confirmed by PCR.

### Preliminary bacteriocin purification

Supernatants of *X*. *euvesicatoria* ME90 (pXV519) cells were collected for size analysis. Bacterial strains were collected from 18 h NB cultures. Cells were removed by filtration. Supernatants from cultures were concentrated by Microcon YM-100 (Millipore, Billerica, MA), Microcon YM-50 units (Millipore, Billerica, MA) with filter cut-offs at 100 and 50 kDA respectively.

### Milk agar protease assay

Proteolytic activity was measured by a diffusion assay in agar plates containing skim milk as a substrate as previously described [36]. Five microliters of each bacterial suspension at OD_600_ = 1.0 was applied to the surface of plates containing 20 mL of 0.5% (w/v) skimmed milk, 2% (w/v) agar and 50 mM Tris hydrochloride, pH 8.0, and allowed to incubate for 24 h at 28°C. Zones of clearing around the colonies caused by proteolytic degradation of the substrate were evaluated.

### Protease fluorescence detection

Protease activity was detected using a Protease Fluorescent Detection kit (PF0100, Sigma, Missouri, USA). In this assay, casein labeled with fluorescein isothiocyanate is used as substrate by potential proteases. Bacteria were grown to OD_600_ = 1.0 in 2 mL of NB. Cells were removed by centrifugation and supernatant was filtered through a 0.22 μm filter. For protease assays, fluorescence of the sample solution was monitored following addition of 20 μl of culture supernatant with a Cytofluor II (Perseptive Biosystems, USA).

### Bioinformatic characterization of *bcn* genes

Each ORF was analyzed for sequence homology (BlastP, http://www.ncbi.nlm.nih.gov/BLAST/), signal peptide (SOSUI, http://harrier.nagahama-i-bio.ac.jp/sosui/sosui_submit.html) and localization (PSORT, http://www.psort.org/).

### Cell fraction activity of Bcn-A

Bacteriocin activity was assessed to determine which fraction(s) contained active Bcn-A protein. *X*. *euvesicatoria* ME90 (pXV519) cells were grown for 18h in NB prior to separation via centrifugation and sonicated using a digital Sonifier® unit model S-150D (Branson Ultrasonics Corporation, Danbury, CT). Supernatant and sonicated cell fractions were assessed for bacteriocin activity by plate assay.

### Homology modeling

Homology models of the full length Bcn-A protein were generated using the iTasser web server [37], and homology models of Bcn-B and Bcn-C were generated using the RaptorX [38] and Phyre2 [39] web servers. The lowest energy homology models generated by each algorithm were visualized, analyzed and figures generated with Chimera [40].

## Results

### Characterization of the Bcn-A region by sequence analysis, subcloning, and mutagenesis

DNA fragments of an 8.0 kb Bcn-A insert from pXV8.0 (Table 1) were subcloned into pLAFR119. Each subclone was expressed in *Xanthomonas euvesicatoria* background, and its ability to produce bacteriocin was tested on nutrient agar using *Xanthomonas euvesicatoria* as an indicator. Only *X*. *euvesicatoria* strains expressing the original 8.0 kb fragment demonstrated Bcn-A activity (Fig 1A). The corresponding region in pXV8.0 contains five genes (ORFA, ORF2, ORF3, ORF4 and ORF5) that may be important in bacteriocin expression and immunity. ORFA is predicted to encode a 1,398 amino acid protein (153.3 kDa) and both the nucleic acid and amino acid sequences showed significant identity to WapA (wall associated protein A of *Bacillus subtilis*), RhsA (rearrangement hot spot elements of *E*. *coli*) and a hypothetical protein from *Coxiella burnetii*. We identified repeat regions in the Bcn-A protein that were similar to those identified as consensus motifs in WapA and RhsA (Fig 1E). The carboxy-terminal half of WapA contains 31 copies of an amino acid repeat sequence called a YD-repeat consisting of the consensus sequence: xxxxGxxxx(Y,F)xYDxxGxxx with a general periodicity of 21 [41]. An almost identical repeat sequence (xxGxxxRYxYDxxGRL{I or T}xxxx) with a similar periodicity was identified in RhsA core elements with repetitions arranged in four blocks of 16, 3, 5, and 3 motifs [42].

The regions of highest sequence identity between Bcn-A and these two proteins lie within the carboxy-terminal 500 residues of Bcn-A. Examination of the Bcn-A sequence in this region reveals the presence of the YD-repeat motif seven times with an approximate periodicity of 24 amino acids. Two other YD-repeat motifs were found earlier in the Bcn-A amino acid sequence at amino acids 431 and 549. The sequences and positions of these motifs are shown in Fig 1E. Similar motifs have also been found in other ligand-binding proteins and are involved in carbohydrate binding [43].

### *ORFA*, *ORF2*, *ORF3*, and *ORF4* are necessary for Bcn-A activity

*ORFA*, *ORF2*, *ORF3*, and *ORF4* mutants were created as described above. Bcn-A activity of these mutants (91–118ΔORFA, 91–118ΔORF2, 91–118ΔORF3, 91–118ΔORF4) was tested against *X*. *euvesicatoria* strain 91–106. Three mutants, 91–118ΔORFA, 91–118ΔORF2 and 91–118ΔORF4 lost complete inhibition activity against *X*. *euvesicatoria* strain 91–106 (Fig 1A and 1B). However, 91–118ΔORF3 had reduced activity compared with the wild-type (Fig 1A). Sequence analyses of ORFs 2, 3, 4, and 5 predict that the ORFs encode proteins of 124 amino acids (13.6 kDa), 100 amino acids (11.0 kDa), 290 amino acids (31.9 kDa), and 145 amino acids (15.9 kDa), respectively. Additionally, ORF4 has an amino-terminal signal peptide and two transmembrane helices, the first spanning amino acids 148 to 170 (VTAVAPPPTPTFQPAILTLGAVL) and the second helix consisting of amino acids 176 to 198 (PAAVSWVSPIMGSIVLAPVLYFA).

### ORF5 is an immunity gene against Bcn-A

A 4.5-kb fragment (pXV4.5) downstream of ORFA was previously found to contain the immunity function against Bcn-A (Fig 1B) [10]. Here, we tested immunity activity of *X*. *euvesicatoria* 91–106 expressing different fragments of the 12.1 kb DNA fragment under a *lac* promoter in pLAFR119 by the deferred antagonism assay to evaluate each immunity candidate. In order to identify the gene(s) responsible for immunity, candidates were gently sprayed onto plates containing 91–118ΔBcnBC (expressing Bcn-A alone), applied to the center of the plate 24 h earlier and incubated for 24 h at 28°C. *X*. *euvesicatoria* transconjugants, 91–106 (pXV12.1), 91–106 (pXV8.0, containing ORF1-5), 91–106 (pXV4.5, containing ORF2-5), 91–106 (pXV8.0 transposon inserted in orf4), and 91–106 (pLAFR119-ORF5) were not sensitive to 91–118ΔBcnBC, while 91–106 (pXV6.2), 91–106 (pXV3.5), 91–106 (pXV4.8), 91–106 (pLAFR119-ORF2), 91-106(pLAFR119-ORF3), and 91–106 (pLAFR119-ORF4) were sensitive (Fig 1B).

**Fig 2:**
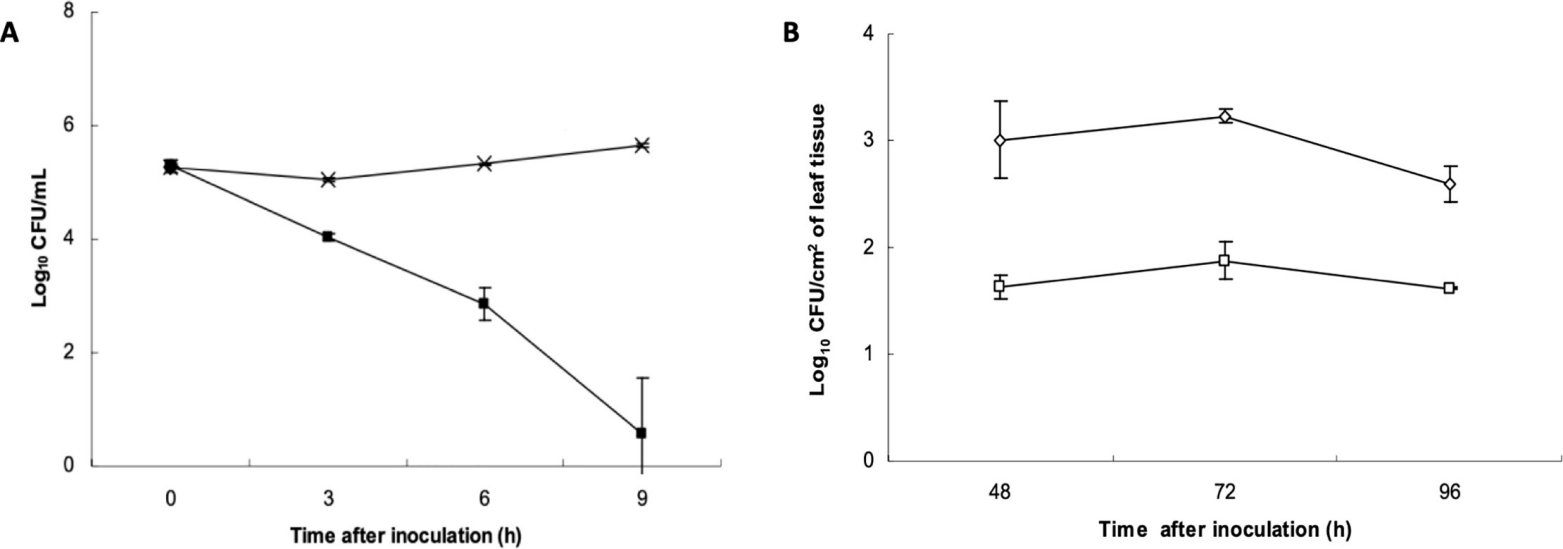
(A) Effect of Bcn-A on the viability of *X*. *euvesicatoria* 91–106 expressing (x) or not expressing ORF5 (ν) *in vitro*. A bacterial suspension of *X*. *perforans* 91–118 MutBC was added to 100 ml nutrient broth to reach a bacterial concentration of 10^7^ CFU/ml. After 7 h incubation, 10^6^ CFU of challenge strains (*X*. *euvesicatoria* 91–106 containing pLAFR119 or pLAFR119*ORF5*) were added. After challenge strains were inoculated, broth was sampled at various times points and plated on nutrient agar amended with nalidixic acid and streptomycin to select for *X*. *euvesicatoria* 91–106. (B). In *in vivo* phyllosphere antagonism assay populations of *X*. *euvesicatoria* strain 91–106 carrying empty vector pLAFR119 (o) or pLAFR119-ORF*5* (ο) were monitored at various time points in leaflets at various time points following infiltration of suspensions adjusted to 5 x 10^7^ CFU/mL into Bonny Best tomato leaflets that were infiltrated 18 h earlier with *X*. *perforans* 91–118 ΔBcnBC suspension adjusted to 5 x 10^7^ CFU/mL. Note that *X*. *euvesicatoria* 91–106 populations not expressing ORF5 declined significantly compared to *X*. *euvesicatoria* 91–106 expressing ORF5. Error bars indicate the standard error.

*In vitro* experiments where *X*. *euvesicatoria* 91–106 (pLAFR119) and *X*. *euvesicatoria* 91–106 (pLAFR119-ORF5) transconjugants were co-inoculated in nutrient broth with 91–118ΔBcnBC to determine sensitivity to Bcn-A. *X*. *euvesicatoria* 91–106 (pLAFR119-ORF5) reached concentrations of 10^5^ to 10^6^ CFU/mL in the presence of Bcn-A, whereas bacterial population of *X*. *euvesicatoria* 91–106 (pLAFR119) co-inoculated with 91–118ΔBcnBC had more than a thousand-fold lower viable cells/ml after 9 h (Fig 2A). In addition, *X*. *perforans* mutant strains 91–118ΔORFA, 91–118ΔORF2, 91–118ΔORF3 and 91–118ΔORF4 all maintained immunity to 91–118ΔBcnBC in deferred antagonism assays. Similarly, in *in vivo* experiments in the greenhouse ORF5 was shown to provide immunity where *X*. *euvesicatoria* 91–106 (pLAFR119-ORF5) was recovered at an average of 1.5 log units higher than *X*. *euvesicatoria* 91–106 (pLAFR119-ORF5) when each was co-inoculated with *X*. *perforans* 91–118ΔBcnBC in leaf tissue (Fig 2B). These results strongly suggest that the product of ORF5 confers immunity to Bcn-A.

### Purification of Bcn-A and role of type II secretion system

Supernatants of *X*. *euvesicatoria* ME90 (pXV519) cells were concentrated with 50 kDa and 100 kDa centriprep centrifugal filter units, (Millipore-Sigma, Burlington, MA). Bactericidal activity was detected in supernatants containing proteins 50 kDa and higher (Fig 1C).

In order to determine if bacteriocin activity was associated with the cell fraction of *X*. *euvesicatoria* ME90 (pXV519), cellular proteins, supernatant and a mix of cellular proteins and supernatant were prepared by sonicating cells. The sonicated cell fraction was filtered through a 0.22 μm filter to eliminate bacteria. Bacteriocin activity was checked by deferred antagonism assay. The cell fraction did not have activity while Bcn-A activity was found in the supernatant (Fig 1D).

An *xpsD* mutant lacking a functional T2SS, *X*. *euvesicatoria* 91-106Δ*xpsD*, was created and a transconjugant containing 91-106 Δ*xpsD* (pXV8.0) was obtained. The transconjugant was tested for Bcn-A activity. Bcn-A activity was observed in the wild-type but eliminated when expressed in the type II secretion mutant (Fig 5A).

### Homology modeling of Bcn-A

The full 1398 amino acid sequence of Bcn-A (i.e., amino acid sequence for *ORFA*) was submitted for homology modeling and the best model is shown in Fig 1F. The model is based upon threading the Bcn-A sequence over the sequences of several proteins of known structure, including: *H*. *sapiens* teneurin 2 (PDB ID: 6FB3), *H*. *sapiens* teneurin-3 (PDB ID: 6FAY), *Photorhabdus luminescens* tripartite toxin (PDB ID: 4O9X) and the BC component of the ABC toxin from *Yersinia entomophaga* (PDB ID: 4IGL). The amino acid sequence identities of Bcn-A to each of these templates ranged from 10% (4IGL) to 22% (6FAY). The teneurin family of proteins are large (~2800 amino acids) membrane anchored proteins responsible for cell-cell adhesion and are thought to have originated from the horizontal gene transfer of a bacterial YD-repeat toxin to an early heterotrophic eukaryote [44]. The central domain of the protein (amino acids 482–1308) is predicted to form a YD-barrel consisting of a 59 beta-strand containing beta-sheet spiraling away from the amino terminus. The amino terminal domains of the protein are predicted to consist of a “plug” on one end of the YD-barrel (amino acids 1–126) and a beta-propeller domain (amino acids 127–481).

In the teneurins and bacterial toxin homologues, the plug domain consists of a fibronectin fold, but the Bcn-A amino acid sequence in this region is not predicted to possess such a fold due to the absence of approximately 140 amino acids compared to the template proteins. Adjacent to the amino-terminal plug is a beta-propeller domain spanning 127–481. In the teneurins, this domain is responsible for interacting with similar proteins and may serve a similar role in Bcn-A. The carboxy terminus of the protein (amino acids 1309–1398) is predicted to cap the YD-barrel and extend into the core of the barrel domain. The Bcn-A homology model suggests that the protein serves as a membrane protein recognition and binding protein, potentially interacting with other subunits as is the case in the ABC toxins. Based upon the model, we hypothesize that Bcn-A interacts with a protein in the outer membrane of *X*. *euvesicatoria* and then binds to the surface of the cell at the amino terminus. The carboxy terminus of the protein may interact with an as yet unknown protein or proteins and puncture the *X*. *euvesicatoria* membrane, thereby delivering the accessory proteins into the target and causing cell death.

### Identification and sequence analysis of Bcn-B and Bcn-C

**Fig 3:**
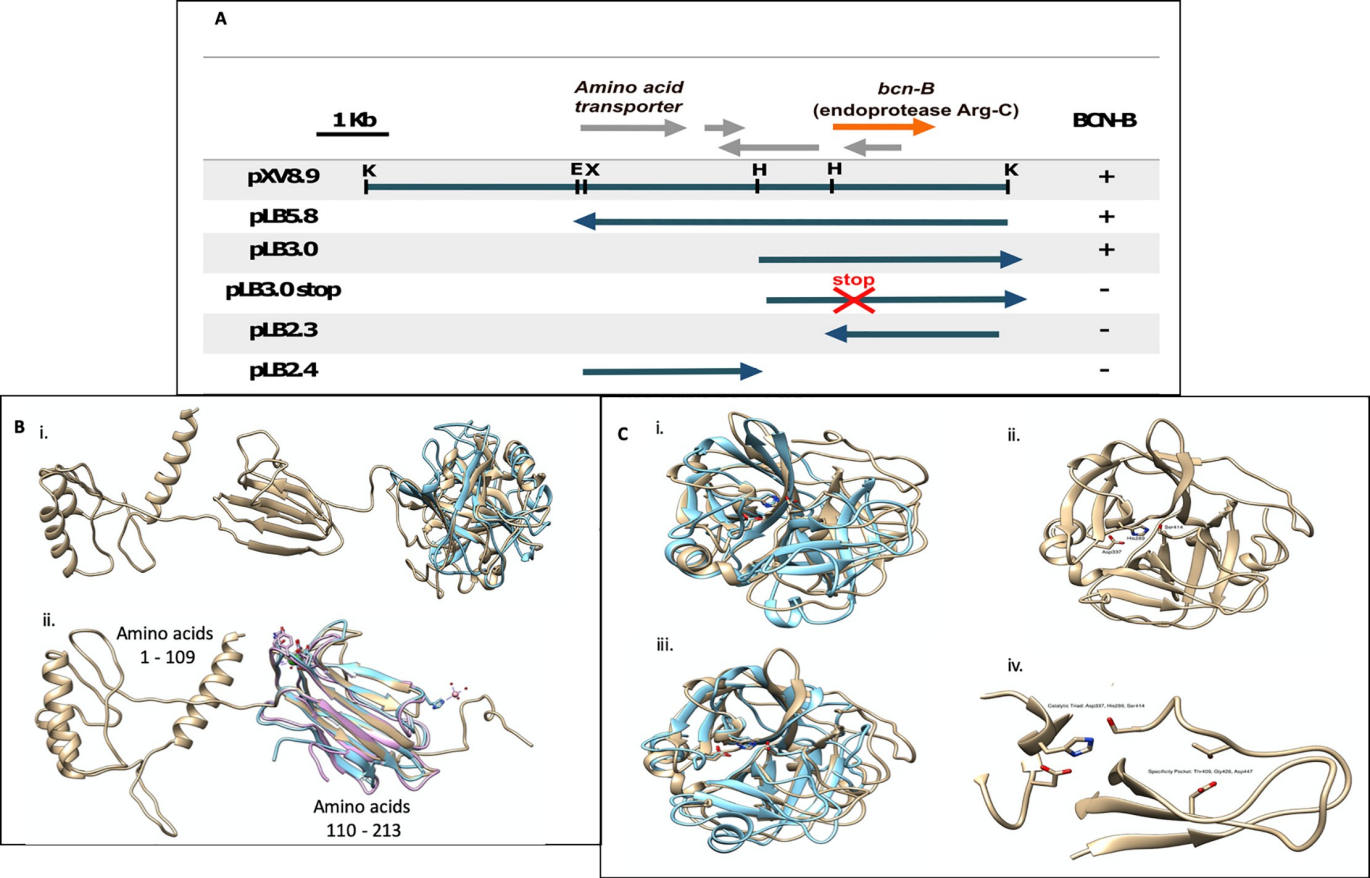
Identification of Bcn-B and characterization by homology modeling. (A) Bcn-B is identified as an endoprotease Arg-C following subcloning of pXV6.0 and mutation by placing a stop codon (TAA) at the N terminus of the putative endoprotease. (B) A superposition of the lowest energy models of Bcn-B is shown. In (i), the models generated by the Phyre2 (blue) and RaptorX (tan) servers are shown. The Phyre2 server did not generate a model structure for amino acids 1 to 213 of Bcn-B, only the carboxy-terminal catalytic domain. In (ii), the model of the two domains predicted by RaptorX to exist at the amino terminus of Bcn-B are shown superposed with the x-ray crystallographic structures of CUB domains from *Homo sapiens* neurophilin-2 (light blue, PDB ID 6GH8) and *H*. *sapiens* TSG-6 (light purple, PDB ID 2WNO). (C) Superposition of homology models of the Bcn-B catalytic domain. In (i), the models generated by RaptorX and Phyre2 are colored tan and light blue, respectively. A superposition of the serine protease domain with the putative catalytic residues shown as sticks is found in (ii). In (iii), the lowest energy RaptorX homology model of Bcn-B is shown in C with the catalytic amino acids (Asp337, His289 and Ser414) labeled and (iv), superposed with trypsin from *Fusarium oxysporium*, the closest homologue identified by a structural search of the PDB (PDB ID: 1XVM).

**Fig 4:**
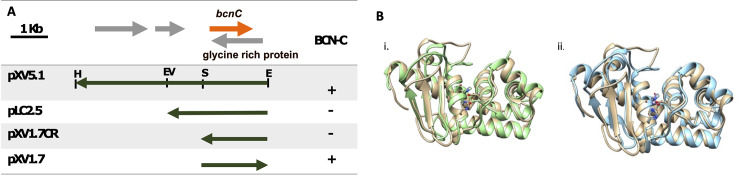
Identification of Bcn-C and characterization. (A) Bcn-C was identified as a metalloprotease following subcloning of pXV5.1 in pLAFR119 followed by expression in *X*. *euvesicatoria* strain 91–106 and using deferred antagonism assay (B)Superpositions of Bcn-C homology models. In (i), the lowest energy homology model of Bcn-C generated by the Phyre2 and RaptorX alogrithms are shown in tan and green, respectively. The putative zinc coordinating residue side chains in each model are drawn as sticks. In (ii), the Phyre2 homology model of Bcn-C (tan) is superposed onto the x-ray crystallographic structure of a zinc metalloprotease from *Grifola fondosa* (blue, PDB ID: 1GE7). The active site zinc ion in the *Grifola fondosa* structure is shown as a sphere with its coordinating water molecules (red).

Previously it was shown that Bcn-B activity was associated with pXV8.9 carrying an 8.9 kb insert [10]. The *Kpn*I*/Eco*RI insert was subcloned into pLAFR3 and designated pLB5.8 (Table 1) [45]. The clone was sequenced and submitted to GenBank (AB302849). Similarly, the *Hind*III*/Eco*RI insert from pXV120 that was previously shown to have Bcn-C activity [10] was subcloned into pLAFR119 and designated pXV5.1 [10]. The resulting transconjugants containing plasmids pLB5.8, with Bcn-B, and pXV5.1, with Bcn-C, inhibited growth of the sensitive *X*. *euvesicatoria* strain, 91–106 (Fig 6A). In order to identify the specific genes responsible for Bcn-B and Bcn-C activities, subclones of different regions of DNA fragments from pLB5.8 and pXV5.1 were ligated into pLAFR119 (Figs 3A and 4A). Each subclone was expressed in *X*. *euvesicatoria* strains ME90 or 91–106 and the ability to produce an inhibition zone was tested on NA media using *X*. *euvesicatoria* strain 91–106 as an indicator. Bcn-B and Bcn-C, which were subcloned to 3.0-kb and 1.7-kb DNA in forward orientation fragments in pLAFR119, respectively, were the smallest fragments that conferred bacteriocin activity (Figs 3A and 4A). Note that Bcn-C was not expressed in the strain containing the 1.7-kb fragment in the reverse orientation (pXV1.7CR) (Fig 4A).

In order to identify the Bcn-B *orf*, directional cloning of the *bcnB* gene was performed in pLAFR119, which contains a *lac* promoter. An ORF with homology to endoprotease ArgC was determined to be responsible for Bcn-B activity based on the lack of activity following creation of a stop codon (TAA) in the forward direction (Fig 3A). Based upon this observation, *bcnB* was determined to consist of 1398 nucleotides (GenBank Accession AB302849) and is predicted to encode a protein of 466 aa (48.5 kDa). Bioinformatic analysis predicts that Bcn-B has non-cytoplasmic signal peptide with an extracellular localization. The predicted amino acid sequence of Bcn-B is more than 98% identical to proteins from several other *Xanthomonas* species such as *X*. *axonopodis*, *X*. *phaseoli*, *X*. *alfalfa*, and *X*. *campestris*.

In order to identify the Bcn-C ORF, directional cloning of the *bcnC* gene was performed in pLAFR119. Given that plasmid pXV5.1 actively expressed Bcn-C without aid of the pL *lac* promoter (Fig 4A), the native promoter was functional. A 1.7 kb fragment of *bcnC* was directionally subcloned in pLAFR119 in both directions. The reverse direction *bcnC* (pXV1.7CR) gave very slight bacteriocin activity compared to under direction of the *lac* promoter (Fig 4A). The *bcnC* gene was determined to consist of 1089 nucleotides (GenBank Accession AB302850) and expected to encode a 362 amino acid (38.2 kDa) protein. Bioinformatic analysis of Bcn-C predicts a non-cytoplasmic signal peptide. The deduced amino acid sequence of Bcn-C shares >97% identity with predicted metalloprotease proteins from *X*. *euvesicatoria*, *X*. *axonopodis pv*. *citrumelo*, *and X*. *citri*.

### Bcn-B and Bcn-C are proteases

**Fig 5:**
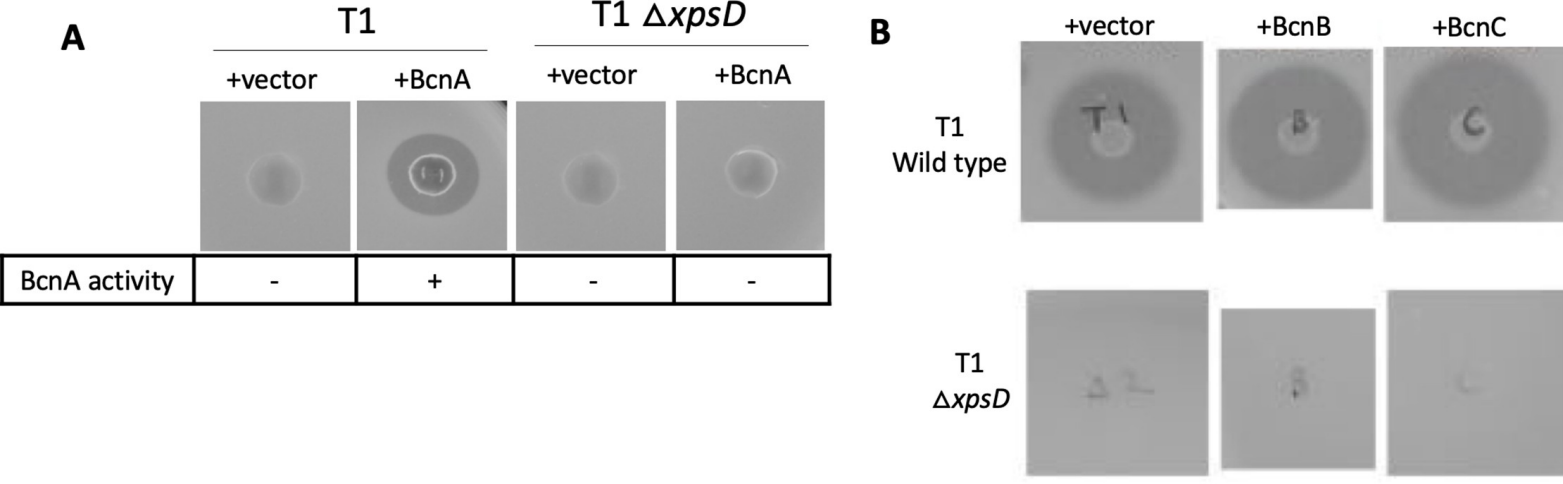
Bcn-A inhibitory activity and Bcn-B and Bcn-C protease activity are associated with the general secretory pathway. (A) 91–106 wild-type and 91–106Δ*xpsD* containing pXV12.1 (BcnA+) were compared for Bcn-A activity using the deferred antagonism assay. (B) 91–106 wild-type and 91–106Δ*xpsD* containing pXV5.8 (+Bcn-B) and pXV5.1 (+Bcn-C) were assayed for protease activity using a milk agar assay after 48 hr. Note that XpsD mutants were negative for activity.

**Fig 6:**
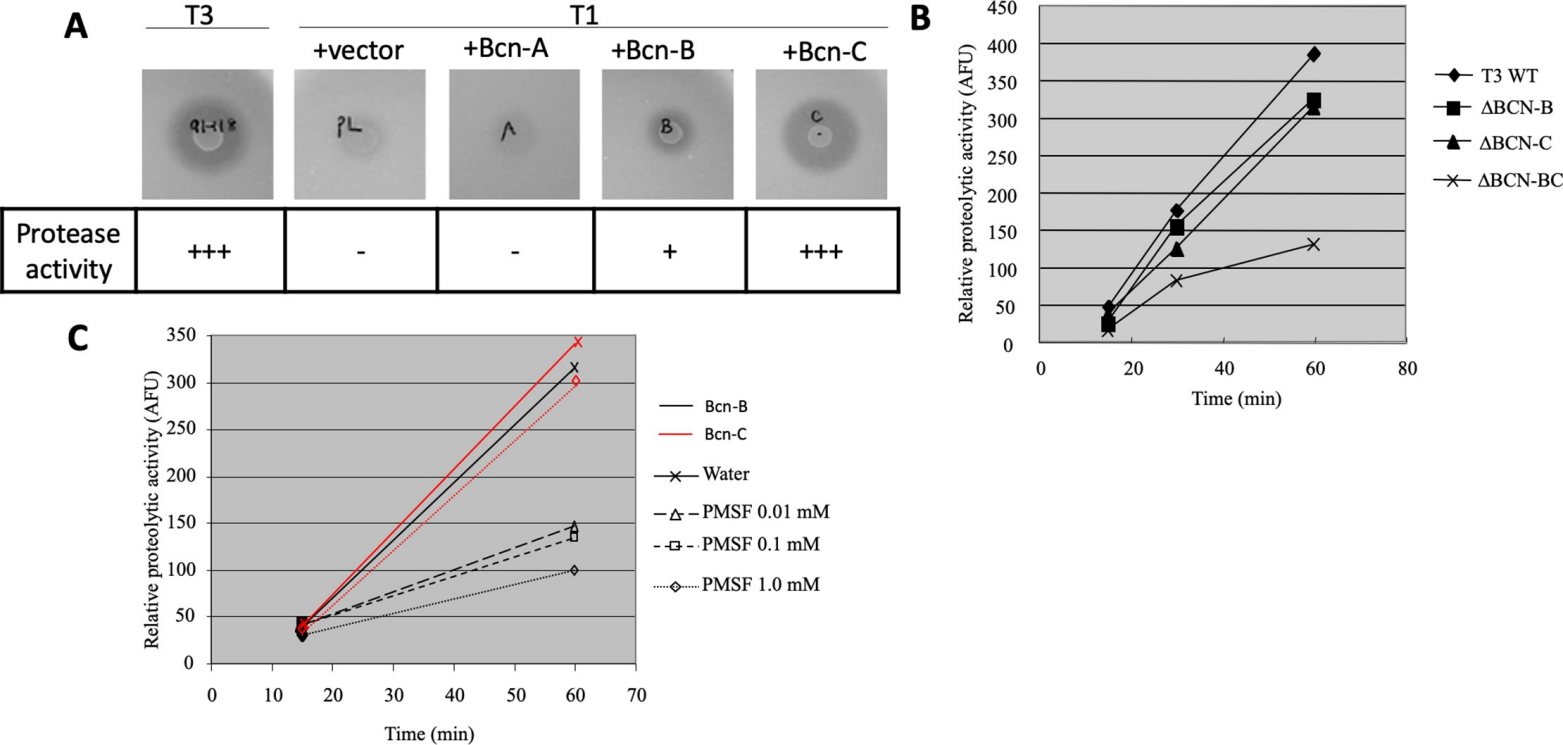
Protease activity of bacteriocins. (A) Protease activity of 91–118 wild-type, and 91–106 conjugated with empty vector or vector expressing Bcn-A, Bcn-B or Bcn-C were shown by a diffusion assay after 24 hr. Zones of clearing around the bacteria due to the degradation of the substrate were observed. Note that the wild-type strain and 91–106 expressing Bcn-B and Bcn-C but not Bcn-A produced clear zones indicating protease activity. (B) Protease activity of 91–118 wild type and mutants were detected by Protease fluorescent Detection kit (Sigma, Missouri, USA). Fluorescence measurements were read by cytofluor II (PerSeptive Biosystems USA). Mutations in Bcn-B and Bcn-C reduced protease activity slightly, whereas the double mutant of both genes resulted in a large drop in protease activity. (C) Inhibition assay with PMSF. Protease activity was determined for ME90 expressing Bcn-B and Bcn-C after treatment with the serine protease inhibitor, PMSF, using the Protease fluorescent Detection kit. Red is Bcn-C and dark lines are Bcn-B. Note that PMSF inhibits Bcn-B at all concentrations but that Bcn-C in not inhibited by PMSF.

As Bcn-B and Bcn-C are homologous to known proteases, proteolytic activity was measured by a diffusion assay in agar plates containing skim milk [36]. Protease activity of *X*. *perforans* ME90 expressing Bcn-A (ME90 (pXV519)) was negative; however, *X*. *perforans* ME90 expressing *bcnB* (ME90 (pXV442)) and *bcnC* (ME90 (pXV120)) produced clear zones (Figs 5B and 6A). A second protease assay was also performed using *X*. *perforans* 91–118 wild-type, 91–118 ΔBcnB, 91–118 ΔBcnC and 91–118 ΔBcnBC, to evaluate the degradation of casein labeled with fluorescein isothiocyanate as the substrate (Fig 6B). Protease assays of *X*. *perforans* 91–118 ΔBcnB and *X*. *perforans* 91–118 ΔBcnC demonstrated a slightly reduced proteolytic activity relative to wild-type. The protease activity of *X*. *perforans* 91–118 ΔBcnBC was very low.

Protease specificity of Bcn-B and Bcn-C were assessed in ME90 expressing Bcn-B, ME90 (pXV442), and Bcn-C, ME90 (pXV120), by measuring protease activity after treatment by the serine protease inhibitor PMSF. Bcn-B activity was inhibited following treatment with PMSF while Bcn-C activity was unaffected, indicating that Bcn-B is a serine protease (Fig 6C) as expected based upon its homology to known serine proteases.

### Homology modeling of Bcn-B

No homologues for the initial 213 amino acids of Bcn-B were identified via BLAST searches of any available database. Submission of the amino acid sequence of Bcn-B to the Phyre2 homology modeling server did not result in any models generated that contained the initial 228 amino acids; however, the RaptorX homology modeling server did generate models for the initial 213 amino acids, indicating that two domains were present in this region (Fig 3B), one spanning amino acids 1 to 109 and another spanning amino acids 110 to 213. No structural homologues could be identified for the first domain; however, the second domain was structurally homologous to CUB domains found in many extracellular proteases (Fig 3B). The degree of amino acid sequence identity between this domain of Bcn-B and other CUB domains from the PFAM database is just 23%, which weakens the confidence of the predicted structure.

Bioinformatic analysis of the amino acid sequence of Bcn-B using BLAST revealed that the carboxy terminus (amino acids 229–465) contains a domain homologous to serine proteases. Submission of the amino acid sequence of Bcn-B to homology modeling servers resulted in models that contained a serine protease domain in that region, with a catalytic triad consisting of Asp337, His289, and Ser414 (Fig 3C-i-iii). Both the Phyre2 and RaptorX algorithms had greater than 90% confidence in the models generated. The closest structural homologue to the lowest energy homology models generated by both algorithm was protease I from *Achromobacter lyticus*. In addition, both algorithms predicted a small S1 pocket (Fig 3C-iv) for the protease domain of Bcn-B, consisting of Gly426 at the base of the pocket and amino acids Asp447 and Thr409 lining the walls of the pocket, suggesting that the specificity of the enzyme is restricted to short chain amino acids.

### Homology modeling of Bcn-C

The Phyre2 algorithm was unable to model the 44 amino acids at the amino terminus of Bcn-C, however both the Phyre2 and RaptorX algorithms were able to confidently model the remainder of the protein. As shown in Fig 4B, the algorithms predicted Bcn-C to adopt an M35-like extracellular metalloprotease fold, a result not unexpected given the greater than 50% amino acid sequence identity to two zinc metalloproteases of known structure. In fact, structural superposition of the Bcn-C homology model generated by the Phyre2 algorithm with the known three-dimensional structure of the zinc metalloprotease from the fungus *Grifola fondosa* reveals that the zinc coordinating residues are predicted to occupy identical spatial positions, with an RMSD of less than 1.044Å for the aligned 108 atom pairs between the proteins.

## Discussion

In this study we identified the genes that are associated with bacteriocins Bcn-A, Bcn-B, and Bcn-C in *X*. *perforans*. For Bcn-A, a five gene locus was identified that contributed to production of and immunity from bacteriocin function. Disruption of ORFA, ORF2 and ORF4 abolished Bcn-A activity based on deferred antagonism assay suggesting Bcn-A is part of a multiple component family of bacteriocins. The protein has significant sequence identity at the amino acid level with an ABC toxin from *Yersinia entomophaga*. This information and the predicted localization of each ORF in the inner and/or outer membrane of the cell suggests that these ORFs make up the necessary parts of a three component system (the toxin, immunity and a mechanism for delivery) of a typical Gram-negative bacteriocin [46]. The first gene in the locus, ORFA, is thought to encode the toxin, and proteins encoded by ORF2 and ORF4 are responsible for possible delivery and processing of Bcn-A (*ORFA* gene product), whereas ORF5 encodes the protein responsible for the immunity function. ORF3 appears to be involved in production of Bcn-A given that there was a mild reduction in antagonism associated with disruption of ORF3. Bcn-A was only detected in supernatants and not at detectable levels in the cell fraction of Bcn-A producing *X*. *perforans* cells, suggesting Bcn-A is activated upon secretion. Therefore, proteins encoded by ORF2, ORF3, and ORF4 appear to play secondary roles such as in transport, modification, or secretion of Bcn-A. An ORF1 was predicted upstream of the Bcn-A genes (Fig 1A) and is annotated as a translocation and assembly module, TamB. TamB is known to form a protein complex involved for assembly of outer membrane proteins in bacteria [47, 48]. However, the *xpsD* deletion mutant lost Bcn-A activity suggesting Bcn-A is secreted via the type II secretion system.

The deferred antagonism assay, growth rate *in vitro*, and *in vivo* experiments strongly suggest ORF5 is responsible for the immunity function. SOSUI predicted ORF5 would be localized to the bacterial inner membrane. This may suggest that ORF5 disrupts or prevents delivery of active Bcn-A into or across the cell membrane or interferes with the function of any extracellular Bcn-A that may bind to *X*. *perforans* cells [49]. However, it is also plausible that the immunity gene functions inside the cell by neutralizing Bcn-A [49]. Col V is one of many known multiple component bacteriocins containing genes encoding the toxin, the immunity function, and the mechanism for delivery. ColV is being used here as our model for basic components of a Gram-negative bacteriocin. In the Colicin V (ColV) secretion pathway of *E*. *coli* in which the immunity protein, Cvi, is delivered into the periplasm where it protects the bacterial cell from ColV activity [50]. Bcn-A is a typical antibacterial toxin in that bacterial strains that contain genes that encode antibacterial toxins also contain genes that encode immunity proteins that protect the producing cell from autointoxication or from toxins produced by other toxin-producing strains [51].

Rhs elements were originally identified in *E*. *coli* as sites that promote recombination [52]. These are composite genetic elements which are repeated in the genome, and are widely distributed among natural *E*. *coli* strains. The large Rhs sequence, which was termed the “core” [42], encodes a conserved N-terminal 1,240 residues of *E*. *coli* Rhs proteins and includes the YD-peptide repeats that define this protein family (Pfam ID:PF03527 and PF05593) [49]. They all share a GC-rich core region of approximately 3.7-kb followed by a variable AT-rich core-extension. This uncharacteristically high GC rich region is believed to have recently been introduced into the *E*. *coli* genome from another organism with high GC content. The largest ORF of these elements spans the core and extension regions and is approximately 4.1-kb in length. This ORF is directly followed by three smaller ORFs (Hill et al., 1994). This core, like the putative *bcnA* gene product, is a high molecular weight, hydrophilic, devoid of a signal sequence protein, and contains 28 copies of an almost identical motif to that found in the C-terminal region of Bcn-A and WapA. A number of secreted ligand binding proteins have been identified as having similar motifs, which are believed to be involved in carbohydrate binding. These include a number of toxins, where the motifs are involved in target recognition [41]. The C-terminal repeating units of ToxA, a toxin secreted by *Clostridium difficile*, are involved in interactions with the oligosaccharide components of receptor molecules on target cells [53]. Several outer membrane proteins have been implicated in bacteriocin binding to target cells [54]; however, only recently has a role for core lipopolysaccharide in bacteriocin binding been demonstrated. Binding of bacteriocin 28, a bacteriocin produced by *Serratia marcescens*, to sensitive cells was blocked in RfaQ mutants which are impaired in core LPS biosynthesis [55]. This may provide a clue as to the role of the putative carbohydrate-binding motifs identified in the *ORFA* gene product. The key elements of these motifs (i.e., a conserved core of aromatic residues followed generally by an asparagine) are present in Bcn-A. This motif has been identified in a discrete C-terminal portion of Bcn-A, which may be suggestive of a domain organization for this protein. Several high molecular weight toxin molecules, including bacteriocins, are organized into discrete domains, in which each has a different function. The domains usually have distinct binding and catalytic functions.

Although initial reports on Rhs elements of *E*. *coli* attributed no known function to these elements, a parallel between these elements and the genetic determinants for bacteriocin production by *E*. *coli* was noted [42]. Colicins are large polypeptides, notably devoid of signal sequences, whose release and immunity are mediated by genes directly downstream of the structural gene. Evidence that they may indeed encode a bacteriocin-like function was obtained when deletion derivatives of the RhsA element lacking the ORFs downstream of the core ORF, were found to impart a toxic effect on *E*. *coli* strains used for routine culturing [43]. A short (72-base pair) ORF, located within the C-terminus of the core ORF, was found to be sufficient to confer toxicity. Similarly, the toxic effects of most Gram-negative bacteriocins are localized in the C-termini of these molecules. Toxicity was only observed after cells had reached the stationary phase of growth. Interestingly, the translation product of dsORF-a1, which lies directly downstream of the core ORF, suppresses toxicity, a structure that mirrors the mechanism of immunity to colicins and immunity to Bcn-A activity. Orf 4 potentially encodes a small protein with some sequence identity to WapA, RhsA, and an insecticidal toxin complex, suggesting that the activity of this protein and Bcn-A may be linked. Genes which confer immunity to bacteriocins are almost always only protective to the bacteriocin with which they are associated. *Carnobacterium piscicola* strain LV17 produces two bacteriocins, carnobacteriocins BM1 and B2. The gene for immunity to B2, was located downstream of the B2 structural gene, and conferred immunity only to this bacteriocin [56]. Rhs elements, like *wapA*, are non-essential to the cells that produce them [57]; however, they have remained highly conserved over a considerable period of evolution. Since they are non-essential for regular cellular functions and are not universally distributed among *E*. *coli* strains, it was proposed that they may play a role in the natural ecology of the cell. Perhaps the finding that they are toxic provides evidence for this. Bcn-A determinants are also not universally distributed among *Xanthomonas campestris* pathovars and Bcn-A negative mutants are viable, indicating a nonessential role for this compound. However, Koskiniemi et al. [49] demonstrated that gram-negative Rhs proteins and distantly related wall-associated protein A (WapA) from Gram-positive bacteria function in intercellular competition. Rhs and WapA carry polymorphic carboxy-terminal toxin domains (Rhs-CT/WapA-CT), which are deployed to inhibit the growth of neighboring cells. These systems also encode sequence-diverse immunity proteins (RhsI/WapI) that specifically neutralize cognate toxins to protect *rhs*^*+*^*/wapA*^*+*^ cells from autoinhibition. Interestingly *orf5*, the immunity gene, does not have homology to any known proteins. Previously *bcnB* was localized to a 5.9 kb fragment [10]. Only two ORFs were found within this fragment that contained homology to genes of known function. One was an amino acid transporter and the other an endoprotease Arg-C. Both genes were isolated and tested for bacteriocin activity. Only fragments containing the intact endoprotease-like gene maintained Bcn-B activity. This ORF was confirmed using an inserted STOP at the 5’ end of the fragment which in turn lost activity confirming that the endoprotease was responsible for the bacteriocin-like activity. Endoprotease Arg-C is a family of serine endoproteases which cleave carboxyl peptide bonds of arginine residues. The enzyme has also been shown to cleave Lys-Lys and Lys-Arg bonds [58].

Bcn-C was previously localized to a 1.7 kb fragment [30]. Two possible ORFs were located within this fragment one in the plus and one in the minus direction. Directional cloning analysis shows that the plus directional ORF was responsible for Bcn-C activity. This gene showed high homology to an extracellular metalloprotease family of genes. Metalloproteases are proteolytic enzymes which use a metal for their catalytic mechanism. Most metalloproteases are zinc-dependent, while some use cobalt or manganese [59].

Bcn-B and Bcn-C were tested for protease activity based on homology data. Our findings show that both Bcn-B and Bcn-C exhibited protease activity; however, Bcn-B produced smaller cleared zones than Bcn-C.
